# When fitness testing becomes personal: achievement emotions and participation orientations in compulsory university physical education

**DOI:** 10.3389/fpsyg.2026.1964318

**Published:** 2026-09-18

**Authors:** Chao Wang, Wenqiang Zheng, Qi Li

**Affiliations:** 1 School of Education and Physical Education, Sichuan Hope Automotive Vocational College, Ziyang, China; 2 Department of Theory and Methodology of Fencing, Russian State University of Physical Education, Sport, Youth and Tourism, Moscow, Russia; 3 School of Outdoor Sports, Guilin Tourism University, Guilin, China

**Keywords:** achievement emotions, assessment, fitness testing, physical education pedagogy, university students

## Abstract

Fitness testing can support learning when pedagogically embedded, but public, graded enactment can expose students' bodies to comparison and judgement. Prior research documents negative experiences but does not explain why similar instances of low performance are interpreted as repairable, personally diminishing, or procedurally unfair. Guided by control-value theory and informed by attribution and self-conscious emotion research, this cross-sectional qualitative critical-incident study examined accounts from 30 undergraduates at three public universities in one western Chinese city. Mandarin semi-structured interviews were analyzed through reflexive thematic analysis and a structured whole-dataset case summary. Fourteen focal incidents were primarily task-focused and repairable, 10 were person- or identity-directed, five were procedure-focused, and one was retained as a boundary case; these counts describe this sample rather than population prevalence. In participants' accounts, residual control, specific attributions, actionable feedback, and retesting accompanied anxiety and preparation. Public identity-laden commentary was described alongside shame, embarrassment, or loss of face and reported concealment, whereas opaque or inflexible procedures accompanied frustration and resistance. These associations were not deterministic: public performance could remain task-focused, and lower-visibility activity could preserve participation. Participants related incident meanings to self-reported post-event actions and anticipated participation; neither was observed or verified longitudinally. The study offers an incident-level account without making causal claims.

## 1 Introduction

Fitness testing occupies an uneasy position in physical education (PE). It can monitor health-related fitness, inform instruction, and encourage physical activity, but may also reduce learning to decontextualised scores. This tension intensifies when testing is compulsory, graded, and conducted before peers. In such settings, students do not submit an artifact separable from its producer; their bodies become the visible objects of assessment. A field-defining critique therefore asked whether fitness testing advances health education or merely classifies bodies (Cale and Harris, 2009). Recent research has moved beyond the binary choice of retaining or removing tests. Students' experiences depend on how testing is embedded in curricula, teaching, feedback, and teacher-peer relations (Alfrey, 2024; Harte et al., 2024; Nolan et al., 2026; Quennerstedt et al., 2025).

This pedagogical shift matters because fitness tests do not carry a single emotional meaning. Public comparison may feel threatening to some students yet manageable to others, and students may report practice, a challenge to the procedure, or withdrawal in relation to a low score. Existing studies document anxiety, embarrassment, shame, reduced enjoyment, and adverse dispositions toward movement (Simonton et al., 2019; White et al., 2025; Yager et al., 2023; Yang et al., 2025). Teachers may mitigate these experiences when testing is embedded in learning and paired with actionable feedback (Alfrey and Young, 2025; Nolan et al., 2026). However, much of this literature reports aggregate associations or broad negative experiences. It therefore does not explain why comparable low performances acquire different emotional targets and participation meanings.

Control-value theory (CVT) provides a starting point because it explains achievement emotions through appraisals of control and value rather than as direct effects of assessment (Pekrun, 2006, 2024). Anxiety is likely when an outcome matters but success remains uncertain; frustration can accompany an obstructed activity; and shame may follow failure interpreted globally and internally. PE research has linked control-value appraisals and emotion-specific experiences with engagement or activity intentions (Harte et al., 2025b; Li et al., 2025; Simonton and Garn, 2019, 2020). Yet variable-level associations do not show why similar instances of low performance remain repairable task problems in some accounts, become procedural injustices in others, or expand into judgements of the person. Attribution theory supplies the locus, stability, and controllability of the stated cause (Weiner, 1985), while self-conscious emotion research distinguishes a negative evaluation of a specific performance from a global evaluation of the self (Tracy and Robins, 2006). These gaps justify examining assessment conditions, control and value, causal attribution, evaluative scope, emotion-specific meaning, and participation orientation as linked analytic dimensions.

These gaps are especially relevant to compulsory university PE in China, where fitness testing occurs within a credit-bearing curriculum while students assume greater responsibility for their physical activity. Chinese research shows that high-stakes school PE assessment can narrow pedagogy toward score production, weaken holistic meanings of PE, and be associated with anxiety and adverse dispositions (Yang et al., 2025). Research with Chinese university students also links teacher support, control-value appraisals, achievement emotions, and physical activity behavior (Li et al., 2025). What remains unclear is how university students interpret particular incidents in which low performance is narrated as repairable information, an obstructing procedure, or a judgement of self or identity, and how they relate those interpretations to different forms of participation.

We address this gap through critical-incident interviews with 30 undergraduates from three public universities in one western Chinese city. We neither propose dignity as a new appraisal category nor infer future behavior from interview intentions. Instead, CVT, attribution theory, and self-conscious emotion research are used together to examine the evaluative target of each incident: a bounded task, an obstructing procedure, or the person and a valued identity. The study contributes, first, a transparent incident-level categorization of how visibility, criteria, feedback, teacher talk, and improvement opportunities entered participants' appraisals and attributions and, second, a differentiated account of how anxiety, self-conscious or social-evaluative emotion, and procedural frustration co-occurred with reported or anticipated preparation, resistance, concealment, or lower-visibility activity.

The study asks:

**RQ1:** Under what reported assessment conditions did students interpret low performance as repairable task information, as the consequence of an obstructing procedure, or as a person- or identity-directed judgement?
**H1** (*post hoc* analytic proposition): Specific, temporary, or modifiable attributions combined with credible guidance or retesting would co-occur with repairable task interpretations; opaque or inflexible arrangements with procedure-focused interpretations; and authoritative comments that extended performance to character, gender, major, or professional suitability with person- or identity-directed judgements.
**RQ2:** How did participants relate these interpretations to anxiety, self-conscious or social-evaluative emotions, procedural frustration, and reported or anticipated participation orientations?
**H2** (*post hoc* analytic proposition): Repairable interpretations would co-occur with anxiety and preparation or guidance-seeking; person- or identity-directed judgements with shame, embarrassment, or loss of face and reported concealment or reduced visibility; and procedure-focused interpretations with frustration, complaint, or resistance.

H1 and H2 were formulated during revision, in response to peer review, as theory-informed, *post hoc* analytic propositions. They were used to re-examine cross-case consistency and discrepant evidence across the complete dataset; they were not preregistered predictions or confirmatory statistical hypotheses.

## 2 Literature review and analytical framework

### 2.1 Fitness testing as pedagogically enacted embodied assessment

Debate about fitness testing has often centered on competing claims concerning measurement, motivation, health promotion, and educational value. Recording performance alone does not teach students how to interpret or develop fitness. It may be counterproductive when scores are detached from meaningful learning (Cale and Harris, 2009). Recent research has sharpened this critique by moving attention from test instruments to their pedagogical enactment. Teachers report using tests for monitoring, motivation, accountability, and fitness education. Yet these educational purposes are not always realized in classroom practice (Harte et al., 2024). Student-voice research similarly shows how testing can be reworked when students' experiences, feelings, and desired forms of learning inform curriculum development (Alfrey, 2024; Alfrey and Young, 2025).

This literature cautions against treating fitness testing as inherently beneficial or harmful. Quennerstedt et al. (2025), for example, distinguish merely testing students from teaching with a test. They locate educational value in the questions, knowledge, and inquiry that testing enables. Conversely, Nolan et al. (2026) show that even resource-rich settings can reproduce public comparison, shame risk, and data collection without actionable feedback. This occurs when performance and talent identification displace educational purposes. Chinese students likewise describe high-stakes PE assessment as overly focused on physical performance and insufficiently inclusive of diverse bodies and activities. They also see it as disconnected from the holistic purposes they attribute to PE (Yang et al., 2025). Together, these studies frame fitness testing as a pedagogical event rather than a neutral instrument.

The embodied visibility of fitness testing makes this event distinctive. Students' experiences of visibility in PE are shaped by lesson content, peer relations, body pressures, previous experiences, and teacher facilitation (Åsebø et al., 2022). Research on changing and showering also shows how bodily exposure can become entwined with shame beyond formal testing (Frydendal and Thing, 2020). Fitness testing intensifies these relations by converting bodily action into a number. That number is compared with criteria or peers and may be attached to grades or public commentary. Assessment and grading research links perceived fairness to understandable criteria and valid knowledge. It also links fairness to credible opportunities to learn and demonstrate capability (Barker et al., 2026; Darabi Bazvand and Rasooli, 2022; Modell and Gerdin, 2022a,b). Yet visibility alone should not be treated as a sufficient cause of shame. A student may perform poorly in public but retain a task-focused interpretation when the result reflects a temporary condition. That interpretation is more likely to remain bounded when a credible route to improvement is available. The analytic question is therefore not merely whether students were seen, but what their visible performance was made to mean.

We treat visibility, comparison, criteria, feedback, retesting, and teacher commentary as assessment conditions rather than a new causal construct. These conditions acquire emotional significance through students' appraisals and attributions. This distinction avoids assigning fixed effects to institutional arrangements when the evidence consists of retrospective student accounts. It also directs attention to pedagogical enactment. A standard may remain stable or change unexpectedly. A score may be announced publicly or discussed privately. Feedback may label the person or identify a specific, improvable feature of performance. Such differences form the context within which students interpret a result.

### 2.2 Control, value, attribution, and self-judgement

CVT proposes that achievement emotions arise primarily from appraisals of control and value (Pekrun, 2006, 2024). Control concerns perceived influence over an activity or outcome. It includes whether effort, strategy, skill, and available support make success attainable. Value concerns the subjective importance of the activity or outcome, including intrinsic interest and instrumental consequences. The theory also differentiates emotions by object focus, valence, and activation. For this study, the crucial point is that a testing event does not directly produce anxiety, shame, or anger. Its emotional meaning depends on what the student perceives to be at stake and what can be done.

This framework has growing relevance to PE. The Achievement Emotions Questionnaire for PE supports emotion-specific measurement in the field (Fierro-Suero et al., 2020). Negative emotions are also associated with distinct behavioral responses rather than uniformly reduced engagement (Simonton and Garn, 2020). Recent research has applied control-value processes to physical activity among university students (Li et al., 2025). Work conducted during fitness-testing lessons has examined anger, anxiety, shame, and other achievement emotions (Harte et al., 2025b). Qualitative analysis can address a complementary question. It can show how participants connect features of a critical incident to the meaning of low performance and to their intended response.

Within CVT's control-value framework, attribution theory offers a finer vocabulary for the locus, stability, and controllability assigned to an outcome (Weiner, 1985). Students may attribute low performance to a temporary bodily state, insufficient preparation, unclear expectations, unstable rules, teacher conduct, or a stable lack of ability. Self-conscious emotion research further distinguishes negative evaluation of a specific action from global negative evaluation of the self (Tracy and Robins, 2006). Shame becomes more plausible when failure is interpreted globally and internally under actual or imagined social evaluation. By contrast, low control and negative value paired with an external attribution to an obstructing procedure may evoke anger or frustration. A specific, modifiable attribution can preserve the possibility of improvement even when the outcome remains important and anxiety-provoking.

This account does not add a third appraisal to control and value. Instead, it specifies the content of control-related attribution in an embodied, socially visible assessment. A score may matter because it affects a grade. The outcome may acquire additional self- or identity relevance when a teacher links it to laziness, masculinity, academic major, or future professional suitability. Such comments can expand the referent of judgement from bounded performance to a valued social identity. We describe participants' interpretation of this expansion as a transition from task-focused interpretation to person-directed judgement. The phrase is an analytic description grounded in attribution and self-conscious emotion theory. It is not a newly validated psychological construct.

### 2.3 From emotional meaning to participation orientations

PE is partly justified by participation beyond the course. Assessment research must nevertheless distinguish observed behavior, retrospective reports of post-incident action, and prospective plans. We use participation orientations for the latter two forms and identify each case as reported or anticipated. Neither form establishes a longitudinal behavioral outcome.

This distinction also prevents exercise volume from becoming the sole outcome. Continuing in private or less visible settings may preserve movement while narrowing its social conditions; it is not necessarily persistence or disengagement. Similarly, short-term training to pass differs from learning that activity is manageable and worthwhile. Transformed fitness-testing pedagogy suggests that agency and meaningful learning require students' feelings and hopes, not compliance alone (Alfrey, 2024; Alfrey and Young, 2025). We therefore examined the form, purpose, and temporal status of participation.

Together, CVT, attribution theory, and self-conscious emotion research sensitized the analysis to valued uncertainty, perceived control, causal locus and stability, evaluative scope, emotion-specific meaning, and action tendency. Assessment conditions were examined as reported contexts rather than as independent variables, and participation was differentiated by temporal status, purpose, and visibility. These linked dimensions generated the two research questions and, during revision, the *post hoc* non-causal analytic propositions above; they did not require a fixed mapping between conditions, emotions, and responses.

## 3 Methods

### 3.1 Design and methodological orientation

The Ethics Committee of the School of Outdoor Sports, Guilin Tourism University approved the study (GLTU-OS-20251023-01). We used a cross-sectional qualitative interview design because the questions concerned how students interpreted particular assessment incidents, not the prevalence of emotions or cause-and-effect relationships. Semi-structured critical-incident interviews enabled participants to reconstruct a memorable episode and describe what occurred before, during, and after it, together with the meanings they attached to performance, comments, and subsequent intentions. Reporting was informed by the consolidated criteria for reporting qualitative research (COREQ; Tong et al., 2007). Analysis followed reflexive thematic analysis (RTA), an interpretive and recursive process in which researchers actively develop themes rather than discover them as objective features of the data (Braun and Clarke, 2006, 2021).

The study was theory-informed and abductive. CVT, attribution theory, and self-conscious emotion research sensitized the analysis to control, value, causal explanation, evaluative scope, anxiety, self-conscious or social-evaluative emotion, and procedural frustration. H1 and H2 were formulated *post hoc* during revision and used to re-examine comparisons; they did not constitute a psychometric or confirmatory model. Because the interview introduction and later prompts named some focal concepts, the themes are not presented as wholly inductive discoveries. Concrete incidents were compared to examine how assessment conditions, appraisals, attributions, emotional descriptions, and participation orientations converged or diverged. Supplementary Appendix A reproduces the interview guide, and Supplementary Appendix B documents the working analytic map.

### 3.2 Setting and participants

Participants were 30 undergraduates from three public universities in a western Chinese city, coded Universities A, B, and C. Eligibility required current enrolment in compulsory PE and recent experience of fitness testing within graded PE. Recruitment used in-class announcements and referrals within PE teaching units. Of 32 eligible students who expressed interest, two declined because of scheduling conflicts; 30 participated and none withdrew. Purposive maximum-variation sampling sought diversity in institution, gender, academic year, major, and prior exercise experience. These dimensions were selected because institutions could broaden the range of reported assessment enactments; gender was relevant to sex-specific tests, embodied visibility, and gendered commentary; academic year varied cumulative exposure to university PE; major could become a valued disciplinary or professional identity and also shape scheduling; and prior exercise experience was theoretically relevant to perceived control and repairability. The dimensions were used to obtain information-rich variation, not as statistical independent variables or demographic quotas.

The sample included 15 women and 15 men, with 12 participants at University A, 10 at University B, and eight at University C. All were first- or second-year students from engineering, medicine, liberal arts, economics and management, and fine arts. Twelve reported structured sport training or regular exercise during secondary school, defined as systematic coaching or sustained exercise at a fixed frequency and duration for more than six months. Supplementary Appendix C provides the anonymised profile. The three sites broadened the range of reported enactments but were not selected to represent Chinese higher education. Participant accounts were treated as perceived assessment configurations rather than audits of institutional policy, and the evidence cannot separate university, course, teacher, or test-item effects.

### 3.3 Data generation

Interviews occurred in quiet, private rooms on the respective campuses, with only the participant and first author present. No course instructor, teaching assistant, or other student attended, and the interviewer had no role in PE grading. Participants received no monetary compensation; after the interview, they received general information about campus physical activity resources unrelated to grades or responses. Interviews were conducted in Mandarin and lasted 45–75 min. At the outset, participants were reminded that involvement was voluntary, confidential, and unrelated to course results. With permission, interviews were audio-recorded and transcribed verbatim.

The guide comprised three modules. Module A documented local testing and grading and reconstructed a memorable, uncomfortable, or high-pressure incident step by step, including who was present, how scores and criteria were communicated, what teachers and peers did, what guidance was available, and whether retesting was possible. Module B explored perceived control, value, attributions, and participants' own distinctions among anxiety, shame, embarrassment, and loss of face; descriptions of frustration, anger, or relief were also considered when present. Module C addressed immediate coping, reported post-incident action, and plans for the next 3 months or the following semester, including preferred activities and settings. Incident reconstruction preceded the most direct emotion questions. However, the opening statement named focal concepts and Module C offered possible participation orientations, making the interviews theory-guided rather than neutral elicitation.

### 3.4 Analysis

Coding and theme development were conducted primarily from the Mandarin transcripts to retain culturally situated and emotional meanings. RTA proceeded recursively through familiarization, initial coding, case-summary construction, candidate-theme development, review against individual cases and the complete dataset, theme definition, naming, and analytic writing (Braun and Clarke, 2021). Within- and cross-case comparisons examined both convergent and boundary accounts, including public performance without global self-devaluation, procedure-focused frustration without self-blame, and low performance accompanied by supportive feedback.

For structured whole-dataset reporting, the eight working families documented in Supplementary Appendix B were consolidated into six linked domains applied to each focal incident: (1) assessment enactment, including visibility, criteria, feedback, teacher or peer commentary, and retesting; (2) control and repairability; (3) causal attribution; (4) value, identity relevance, and evaluative scope, distinguishing a bounded task or procedure from the person or a valued identity; (5) emotional meaning; and (6) action and participation orientation, including temporal status and visibility. Boundary evidence was retained as a cross-cutting check. An incident was summarized as repairable when the participant identified a specific, temporary, or modifiable cause and a credible next step; as person- or identity-directed when performance was interpreted as commentary on character, worth, gender, major, or professional suitability; and as procedure-focused when obstruction was located mainly in criteria, timing, rules, or access to review. Multiple codes could co-occur. The primary pathway was assigned from the dominant causal and evaluative target in the complete account, while secondary codes and discrepant evidence were retained.

After theme development, a 30-row case-summary matrix was compiled from the Mandarin materials, and these decision rules were used to summarize primary-pathway coverage. Mixed cases retained secondary codes, and one case was left as a boundary case rather than forced into a pathway. The primary assignments and supporting evidence were reviewed in author-team discussions. Where readings differed, the team revisited the complete case summary, recorded alternative interpretations, and retained secondary or boundary status when a single assignment could not be justified. These discussions functioned as critical dialogue rather than independent reliability coding. The post-theme content summary did not constitute inferential statistical analysis. Case counts and participant identifiers are reported as descriptive evidence coverage only; they do not estimate prevalence, effect size, or statistical generalisability. Theme development preceded English reporting. Selected Chinese source material and English renderings are available in Supplementary Appendix D.

### 3.5 Reflexivity and qualitative quality

The interviewer's lack of grading authority reduced but could not remove the evaluative relationship surrounding discussion of PE. Reflexive memos recorded prior assumptions, interpretive shifts, and alternative explanations. Conceptual sufficiency was judged through depth and variation rather than a numerical saturation rule. Analytic quality was supported by incident-level reconstruction, within- and cross-case comparison, explicit boundary cases, transparent acknowledgment of prompted concepts, and separation of reported or anticipated participation from researcher-observed behavior.

All participants provided written informed consent. Identifying details were minimized, and the university codes refer only to the study sample. Nevertheless, combinations of major, year, gender, and distinctive incidents may create recognition risks within local settings. The supporting profile should therefore be read as contextual information rather than as a basis for identifying individual cases.

## 4 Findings

Sections 4.1–4.3 address RQ1 and the *post hoc* H1 proposition, and Section 4.4 addresses RQ2 and the *post hoc* H2 proposition. The structured whole-dataset summary identified 14 incidents primarily interpreted as repairable task problems, 10 as person- or identity-directed judgements, five as procedure-focused judgements, and one boundary case. Table 1 lists every primary assignment. These descriptive counts indicate evidence coverage within this sample; they are not prevalence estimates, mutually exclusive participant types, or confirmation of an *a priori* causal model. Secondary codes were retained when accounts crossed pathways. The overall pattern broadly aligned with the *post hoc* propositions, but the boundary and contrasting cases prevented a deterministic interpretation.

**Table 1:** Structured case coverage across 30 reported critical incidents.

Assessment conditions in the account	Primary interpretation or attribution	Reported emotional meaning	Reported or anticipated orientation	Descriptive case coverage
Valued outcome; specific or temporary difficulty; credible guidance, adaptation, or retesting	Performance is uncertain but repairable	Anxiety, concern, regret, or self-criticism without global self-devaluation	Preparation, guidance-seeking, adaptation, or stated continuation	*n* = 14: P2, P5, P7, P12, P13, P16, P19, P21, P24, P26–P30
Social exposure with person- or identity-directed commentary or labeling	Assessment is interpreted as judging the person or a valued identity	Shame, embarrassment, loss of face, or social-evaluative discomfort	Concealment, avoidance, reduced intention, or narrower acceptable settings	*n* = 10: P1, P3, P4, P6, P10, P11, P15, P20, P23, P25
Opaque, unstable, inflexible, or insufficiently explained procedure	Difficulty is attributed primarily to an obstructing external arrangement	Procedural frustration, anger, uncertainty, or feeling unfairly treated	Complaint, negotiation, resistance, or minimum compliance	*n* = 5: P8, P9, P17, P18, P22
Public difficulty without a clear person/identity or procedure-directed interpretation	Boundary account retained rather than forced into a pathway	Anxiety and mild shame-related embarrassment	Compliance-oriented walking and avoidance of attention	*n* = 1: P14

Primary assignment was based on the dominant causal and evaluative target in each complete account; secondary codes were retained for mixed cases. “Reported or anticipated orientation” refers to participants' stated post-incident actions or future intentions, which were not observed or verified longitudinally. Counts describe evidence coverage in this sample, not prevalence or causal effects.

### 4.1 RQ1/H1: repairable uncertainty and task-focused interpretation

Anxiety was most clearly described when a result mattered but improvement still appeared possible. These accounts involved uncertainty about passing, retesting, ranking, physical readiness, or preparation time. The experience remained task-focused not because pressure was absent, but because the participant attributed the result to a specific or temporary condition, identified a modifiable performance problem, or described a credible route to another attempt.

P2, who exercised regularly, began an 800-meter test expecting to perform well. During menstruation, she ran below her usual level and was compared publicly with classmates. She described mild anxiety and feeling wronged rather than shame, attributing the result to a temporary bodily condition rather than incapacity.

P2 explained the bodily condition to the teacher and reported adding targeted running to her preparation. Public comparison was therefore present, but the wider account remained performance-specific: prior competence and a temporary explanation bounded the meaning of the result and left a feasible response.

P13 described a different configuration after missing the sit-and-reach criterion on her first attempt. The teacher discussed the result privately, demonstrated a stretching method, and offered a retest. She reported concern about passing but no shame, and she practiced the recommended technique. Privacy co-occurred with actionable information and another opportunity; the account does not show that privacy alone produced the task-focused interpretation.

Across the 14 primarily repairable incidents, the delivery format varied. P12 missed a standing long-jump standard by one centimeter, sought technical guidance, and planned daily practice. P16 attributed a disappointing 1,000-meter time to pacing and added steadier running to team training. P26 linked process-oriented encouragement with a daily walking or brisk-walking routine, while P28 and P29 described low-intensity, context-sensitive guidance. The common feature was a specific or modifiable interpretation with a credible next step, not a single level of visibility or pressure.

Contrasting cases sharpened this interpretation. P2 showed that visibility and comparison could coexist with mild anxiety and targeted preparation without global self-devaluation. P30 missed the threshold for an excellent rating while managing a minor injury; he described mild anxiety and regret, continued basketball training, and added middle-distance work after recovery. Thus, ‘repairable' denotes the participant's specific and modifiable interpretation of the task, not an objectively easy event or an absence of negative emotion.

### 4.2 RQ1/H1: when performance became a judgement of the person or identity

The 10 primarily person- or identity-directed incidents involved more than uncertainty or low scores. Participants interpreted a specific bodily performance as commentary on character, effort, gender, academic identity, professional suitability, or social adequacy. In the clearest cases, the comment came from the authorized assessor; peer attention or ridicule sometimes intensified its social visibility. The defining feature was the perceived expansion of judgement beyond the immediate task.

P1 reconstructed a pull-up test conducted before classmates. Repetitions were counted publicly, and he could not complete one. The teacher then said, “An engineering boy with physical fitness this poor,” after which some classmates laughed.

The comment identified neither technique nor preparation. P1 interpreted it as a judgement of his adequacy as a male engineering student rather than a bounded pull-up result. In the wider account, peer laughter accompanied shame and a wish to remain unnoticed, and he later reported standing at the back of PE lessons.

P4 reported a similar shift after becoming dizzy during an 800-meter run. The teacher asked publicly, “With this little stamina, how will you become a doctor?” P4 interpreted the remark as questioning her suitability for medicine rather than commenting only on running. What was at stake therefore extended from a course result to a valued professional identity.

Across the remaining cases in this pathway (P3, P6, P10, P11, P15, P20, P23, and P25), repeated public failure, peer attention, or comments about effort, appearance, major, or professional adequacy were described alongside shame, embarrassment, loss of face, or a wish to become less visible. P6, for example, explicitly connected repeated public attempts and an effort-related label with shame, zero exercise intention, and discomfort on seeing the track. P20 contained a secondary procedural concern about scheduling, but the dominant evaluative target in his account was the teacher's identity-laden judgement of his commitment to art and fitness. Not every imposed label became a stable global belief, and the intensity and reported orientation varied across cases.

Visibility increased the social salience of these judgements but did not distinguish pathways by itself. P2's public result remained task-focused because she interpreted it as temporary and non-diagnostic, whereas P1 and P4 interpreted authorized comments as extending the incident to gendered or professional identity. Anxiety concerned an uncertain valued outcome; shame, embarrassment, and loss of face concerned how the self or social identity appeared to be judged. Concealment and reduced visibility were analyzed separately as action or participation orientations.

### 4.3 RQ1/H1: when the procedure, not the self, was blamed

A third pattern redirected negative evaluation toward the assessment procedure. Five primary cases described frustration alongside complaint or resistance when difficulty was attributed to changing standards, fixed testing times, unclear rules, absent guidance, or limited routes for review. Perceived control was low, but the dominant attribution did not become a judgement of deficient ability or personal worth.

P8 prepared for a sit-and-reach criterion that was subsequently changed without what she considered adequate notice. She rejected shame, described anger and unfairness, and attributed the disappointing outcome to a broken link between preparation and assessment. She joined classmates in challenging the decision and reported continuing her usual physical activity.

P18 similarly directed frustration toward inflexible timing and limited routes for redress. P9 and P22 described conflicts between fixed testing or retesting times and academic or placement commitments, while P17 emphasized an unexplained pass/fail outcome and absent guidance. These accounts did not present complaint as a refusal to accept demanding standards. Rather, participants questioned whether the procedure allowed preparation or legitimate commitments to count. Secondary embarrassment appeared in some cases, but the dominant causal target remained the procedure.

These accounts qualify any linear claim that low control is followed uniformly by withdrawal. Temporary or modifiable attributions co-occurred with anxiety and preparation; procedure-focused attributions with frustration and challenge; and person- or identity-directed judgements with self-conscious or social-evaluative emotion. The pattern reflects differences in the stated attribution and evaluative target, not a verified temporal or causal sequence.

### 4.4 RQ2/H2: participation differed in timing, purpose, and setting

Participants related incidents to reported post-event actions and intentions for the next 3 months or the following semester. In relation to the *post hoc* H2 proposition, their accounts differed not only in exercise volume but also in purpose, setting, visibility, and whether participation centered on learning, passing, self-protection, or resistance. These were interview reports, not observed or longitudinally verified behaviors.

Task-focused interpretations co-occurred with preparation or guidance-seeking across the repairable pathway. P2 reported adding running, P12 planned technique practice, and P16 integrated pacing work into an existing team routine. Some accounts concerned learning, whereas others described short-term attempts to pass or restore a preferred grade. Reported or planned activity could therefore signify developing competence or controlled compliance.

In person- or identity-directed accounts, participants often reported concealment, avoidance, or a narrower range of acceptable settings. P1 described standing at the back and avoiding notice in later lessons; P4 anticipated solitary slow walking rather than group running; and P6 reported no exercise intention and discomfort associated with the track. These action and participation orientations were described alongside self-conscious or social-evaluative meanings; they were not emotions in themselves.

P14 was retained as the single boundary case. He reported strong anxiety and mild shame-related embarrassment when publicly prompted and overtaken during a 1,000-meter test, but did not clearly interpret the event as a judgement of character or identity or primarily as a procedural injustice. He reported largely compliance-oriented walking after the incident. Leaving this case unforced makes the limits of the three-pathway summary visible.

Lower visibility did not always indicate withdrawal. P26 linked process-oriented encouragement with a plan for daily campus walking or brisk walking. P29 described individualized guidance connecting movement with the physical demands of drawing and anticipated stretching and indoor walking. These accounts show that lower-exposure activity can support contextually relevant adaptation; setting alone does not determine meaning. Table 1 summarizes all primary case assignments and their typical emotional and participation patterns.

## 5 Discussion

### 5.1 From low performance to person-directed judgement

Low fitness performance did not carry a fixed emotional meaning. Across the whole-dataset summary, participants interpreted it primarily as bounded and repairable task information, as the consequence of an obstructing procedure, or as a judgement of the person or a valued identity; one account remained outside these pathways. This pattern broadly aligned with the *post hoc* H1 proposition but does not constitute a statistical test. It extends work locating fitness testing's educational and emotional significance in pedagogical enactment rather than in the instrument alone (Alfrey, 2024; Harte et al., 2025a; Nolan et al., 2026; Quennerstedt et al., 2025) by showing how reported conditions entered contrasting incident interpretations.

The evidence does not support a direct sequence from public testing to negative emotion. Visibility exposed performance to social attention but did not distinguish whether the participant's account remained task-specific, blamed an obstructing procedure, or extended judgement to the person. Privacy was not inherently educative either: in supportive accounts, it accompanied usable feedback and a credible opportunity to improve. The sharper distinction concerned evaluative scope. A result remained task-focused when participants named a specific, modifiable cause and a feasible next step; it became person- or identity-directed when an authorized comment linked bodily performance to character, gender, academic identity, or professional suitability.

This distinction contributes a PE-specific account to assessment research on identity formation (Nieminen, 2025; Nieminen and Yang, 2024). Because the body is both the performance medium and visible assessment object, feedback may be interpreted as shifting from what happened to what the performance implied about the student. The educational issue is not whether fitness data may be evaluated, but whether evaluation remains specific enough to support learning. The present qualitative evidence also complements variable-level research linking appraisals and emotions with physical activity intentions (Li et al., 2025; Zimmermann et al., 2021): it supplies incident-level meaning and boundary cases but cannot confirm mediation or behavioral effects.

### 5.2 Differentiating anxiety, self-conscious emotion, and procedural frustration

The findings align with CVT's premise that appraisals, rather than assessment events alone, differentiate achievement emotions (Pekrun, 2006, 2024). In repairable accounts, passing, grades, reputation, or identity mattered and success was uncertain, but practice, guidance, pacing, adaptation, or retesting preserved residual control. Anxiety could therefore co-occur with preparation. This is consistent with PE research showing that negative activating emotions do not map uniformly onto disengagement (Fierro-Suero et al., 2023; Simonton and Garn, 2020), while the boundary cases show why no invariant pathway should be inferred.

Attribution theory adds precision by locating the stated cause and its stability or modifiability (Weiner, 1985). Self-conscious emotion research adds evaluative scope: a specific performance can be judged negatively without extending that judgement to the whole self (Tracy and Robins, 2006). Temporary bodily states, pacing, or technique remained bounded; opaque procedures redirected evaluation toward the system; and identity-laden commentary widened what appeared to be judged. Shame, embarrassment, and loss of face were not treated as interchangeable, and the evidence does not show that every external label became a stable global self-belief. These distinctions explain the added value of combining CVT with attribution and self-conscious emotion research rather than proposing a new appraisal construct.

Procedure-focused accounts mark a further distinction. Participants located obstruction in changing criteria, fixed timing, absent guidance, or limited review and described frustration alongside complaint, negotiation, resistance, or minimum compliance. Because some cases also contained embarrassment or identity-related comments, the structured summary used the dominant causal and evaluative target while retaining secondary codes. Across all pathways, the reconstruction is an interpretive association among reported conditions, appraisals, emotional meanings, and stated orientations—not a verified temporal sequence or causal mechanism.

### 5.3 Cross-national and socio-cultural interpretation

The accounts both converge with and qualify findings from other national contexts. Research in England and Australia has linked performance-oriented testing, public comparison, and limited actionable feedback with anxiety, embarrassment, or adverse dispositions (Harte et al., 2025a,b; Yager et al., 2023), while Danish research shows that bodily visibility can acquire shame-related meanings beyond formal testing (Frydendal and Thing, 2020). The present accounts likewise emphasized visibility and teacher-peer relations, but public performance alone did not separate the pathways. In the Chinese compulsory, credit-bearing context, teacher authority and comments connecting fitness with gender, major, or professional suitability made some judgements especially consequential. Participants' language of losing face should not be treated as a uniquely Chinese trait or as evidence that culture caused the response. Similar social-evaluative processes appear internationally; what varied here was the institutional and cultural form through which authority, identity, and comparison were articulated. The findings also extend Chinese research on high-stakes PE assessment and self-directed fitness testing (Yang et al., 2025; Zhang et al., 2026) by showing both identity-threatening and supportive, task-focused enactments within university PE.

### 5.4 Participation orientations and pedagogical implications

The pattern broadly aligned with the *post hoc* H2 proposition: repairable interpretations accompanied reported or planned preparation; person- or identity-directed judgements accompanied self-conscious or social-evaluative emotion alongside concealment or reduced visibility; and procedure-focused interpretations accompanied complaint or resistance. These associations complement research connecting appraisal and achievement emotion with physical activity intentions among university students (Li et al., 2025), but the cross-sectional interview design cannot show that an emotion caused a later activity pattern.

Some participants reported or anticipated moving in private, solitary, or less visible settings. Such choices may represent adaptive self-protection rather than withdrawal from movement, but they may also indicate that a course has narrowed the social conditions under which activity feels acceptable. Conversely, intensive preparation for a score may demonstrate compliance without broader learning. Performance-oriented testing can displace educational purposes and generate data without usable guidance (Harte et al., 2025a; Nolan et al., 2026; Yang et al., 2025). Accordingly, participation should be interpreted by purpose and setting as well as by amount.

At the teacher level, scores and feedback should usually be communicated privately, comments should identify a specific feature of technique, pacing, preparation, or strategy, and students should receive a feasible next step. At the course level, criteria, accommodations, retesting, and review procedures should be stable, written, and explained before testing; fitness data should be integrated with interpretation, practice, and reassessment. At the departmental level, staff development should address person- and identity-labeling, while schedules, facilities, and workload should make privacy, guidance, and reassessment practicable. These applications align with process-oriented, student-voice, and inclusive assessment (Alfrey and Young, 2025; Tai et al., 2023). Their effectiveness should be evaluated through observation and longitudinal follow-up rather than assumed from the present accounts.

Implementation involves real tensions. Privacy must coexist with class organization; flexible retesting and reasonable adjustment require time, staffing, and facilities; and process-sensitive assessment must still produce credible institutional decisions. A defensible response is not complete individualization but transparent judgement and meaningful opportunities to learn. Reform therefore cannot rest on individual teacher goodwill alone: departments need procedures and resources that make preparation, private and actionable feedback, review, and reassessment workable.

### 5.5 Limitations and future research

Several limitations qualify this contribution. First, the cross-sectional retrospective design cannot establish causal or temporal relationships. Critical-incident prompts likely privileged memorable, uncomfortable, or high-pressure events and invited reconstruction through hindsight. The guide named focal emotions and offered prospective participation categories, creating demand characteristics and the possibility of socially desirable responses—for example, presenting oneself as diligent, unfairly treated, or resilient. Private interviews and the interviewer's lack of grading authority may have reduced, but could not eliminate, evaluation apprehension, interviewer effects, or response bias. In-class announcements and peer referrals may also have produced self-selection. The theory-informed categories and case counts therefore describe the available accounts, not unprompted category prevalence.

Second, the 30 participants came from one western Chinese city and only the first two academic years. The sample cannot estimate prevalence, support population generalization, or separate university, course, teacher, test-item, gender, major, and prior-experience effects. Participant reports were not triangulated with lesson observation, assessment documents, teacher accounts, or behavioral measures. English reporting involved interpretive translation choices, and reported post-incident actions and future plans were neither observed nor followed longitudinally. Future studies should combine interviews with observation and institutional records, recruit across regions and year groups, and follow participation over time. Intervention research could then compare private actionable feedback, stable criteria, process-focused goals, structured review, and retesting with score-only enactments.

## 6 Conclusion

Participants did not describe public or low performance as carrying a fixed emotional meaning. Across their accounts, specific and repairable interpretations co-occurred with anxiety and preparation; procedure-focused attributions with frustration and resistance; and person- or identity-directed judgements with shame, embarrassment, or loss of face alongside concealment or narrower participation settings. These are cross-sectional, self-reported associations, not causal effects or observed longitudinal pathways. The pedagogical implication is nevertheless clear: performance information should remain specific, actionable, and separate from judgements of personal worth. Compulsory PE is most defensible when testing supports interpretation, practice, fair review, and reassessment rather than publicly diagnosing the student.

## Data Availability

The original contributions presented in the study are included in the article/Supplementary material, further inquiries can be directed to the corresponding author.
