# Supplementary material for: When fitness testing becomes personal: achievement emotions and participation orientations in compulsory university physical education
Source: Front Psychol. 2026 Sep 18;17:1964318. doi: 10.3389/fpsyg.2026.1964318 (PMC13630932; doi:10.3389/fpsyg.2026.1964318)
Supplement: Supplementary file 1 [file Supplementary_file_1.docx]

**Supplementary Appendices**

# **Supplementary Appendix A. Semi-structured interview guide used in data generation**

**Transparency note.** This appendix reproduces the theory-guided interview structure used in the study. The opening named control, value, anxiety, shame, and dignity, and Module C offered examples of possible participation orientations. The revised article therefore does not present its focal concepts as wholly inductive discoveries. The guide is retained here rather than retrospectively rewritten as a neutral instrument.

**Opening.** Thank you for joining. This study explores how students experience fitness testing and grading in university PE, how they interpret these experiences in relation to control, value, anxiety, shame and dignity, and how these meanings may shape intentions for exercise beyond the course. The interview will take about 60 minutes. With your permission, it will be audio-recorded for transcription and analysis only. Participation is voluntary; you may skip any question or stop at any time without consequences.

**Warm-up.** Have you exercised in the past week? What kinds of activities do you usually prefer?

## ***A1. Participant background***

What year are you in, and what is your major? Is the PE course required or elective? How often do you exercise in a typical week? Before university, did you have structured sport training or regular exercise habits? How would you describe your current fitness or physical condition, and why?

**Probe:** Do you generally feel confident in PE contexts? What shapes that confidence?

## ***A2. Module A: Assessment context and critical-incident reconstruction***

What fitness tests are included in your PE course? How is your grade calculated? How clear are the criteria? How did you learn them? How important is the grade or testing outcome to you?

Please recall one fitness test or grading experience that stands out as memorable, uncomfortable, or high-pressure. What happened step by step? Was the situation public or private? Were peers watching? Were scores announced or posted? What comments did the teacher make? What did peers do? Which feature triggered most pressure or discomfort, and why?

**Probe:** Did the event feel outcome-only or process-recognising? Was there guidance before or after evaluation?

## ***A3. Module B: Control-value appraisals and emotional meanings***

Before the test, how confident were you about performing well? What informed that judgement? What did the result depend on? Which parts felt controllable, such as effort, preparation or technique, and which felt uncontrollable, such as body condition, fixed standards, teacher practices, environment or luck? What does the test score or grade mean to you personally? If the result is poor, what is the most salient consequence?

Does the assessment feel more like evaluating physical ability or evaluating you as a person? Did you experience anxiety before, during or after the test? Did you experience shame, embarrassment or humiliation? What triggered it? How would you distinguish anxiety from shame in your own experience? Did the situation threaten or protect your sense of dignity? What actions did these feelings push you towards?

## ***A4. Module C: Coping and exercise intentions beyond the course***

When you feel anxiety or shame related to testing or grading, what do you typically do? How effective are these strategies in the short term and longer term? After the experience, did your exercise intentions move towards sustained exercise, short-term compensatory training, reduced exercise, avoidance or a shift to less visible activities? What is your exercise plan for the next three months or next semester? If the assessment design changed, how might your emotions and exercise intentions change? What feels distinctive about your university's PE assessment culture or arrangements?

## ***A5. Closing***

If you could change one thing about fitness testing or grading in university PE, what would it be and why? What advice would you give teachers or administrators to reduce unnecessary anxiety or shame while still supporting long-term exercise participation? Is there anything important we did not ask?

# **Supplementary Appendix B. Analytic map and interpretive pathways**

## ***B1. Purpose and methodological status***

The analysis was theory-informed and abductive. Control-value theory, attribution theory, and self-conscious emotion research sensitised attention to control, value, causal explanation, anxiety, shame-related meanings, and anger or frustration, while incident-level comparison was used to examine where accounts converged with or complicated those expectations. The following tables document a working analytic map used for reporting. They are not a fixed codebook, a measure of theme frequency, or evidence of inter-coder reliability.

Because the interview guide introduced focal concepts, the revised analysis makes three safeguards explicit:

1. It does not claim that dignity, emotional categories, or participation-orientation categories emerged without prompting.
2. It retains boundary accounts in which public performance did not involve shame and low control was directed towards an unfair procedure rather than the self.
3. It distinguishes reported post-incident actions, future intentions, and researcher-observed or longitudinal behaviour.

## ***B2. Working analytic map***

| **Analytic family** | **Guiding question** | **Illustrative indicators** | **Role in theme development** |
| --- | --- | --- | --- |
| Assessment conditions | How was the incident pedagogically enacted? | Visibility; peer comparison; score communication; criteria; teacher talk; feedback; retesting; instructional support | Located the social and instructional context without treating it as an independent causal construct |
| Control and repairability | Did the student see a credible way to affect or improve the outcome? | Preparation; technique; pacing; temporary bodily condition; guidance; repeat attempt; blocked effort–outcome link | Distinguished valued uncertainty with residual control from experienced helplessness |
| Attribution | Where and how broadly was difficulty located? | Temporary/specific condition; own preparation; unstable rule; teacher conduct; stable ability; global self | Distinguished task-focused, system-directed, and person-directed interpretations |
| Value and identity relevance | What made the outcome matter? | Pass/grade; ranking; reputation; gender; academic major; future professional suitability | Identified when a task result acquired broader self or identity meaning |
| Emotional meaning | What emotion did the participant name or describe, and what was its target? | Anxiety/worry; shame/self-devaluation; embarrassment or loss of face; humiliation; anger/frustration; regret; relief | Avoided treating negative affect as a single category |
| Action tendency | What did the emotion orient the participant to do at the time? | Practise; seek guidance; explain; complain; hide; leave; avoid; reappraise | Connected meanings with immediate coping without inferring causality |
| Participation orientation | Did the participant report an action after the incident or state a future plan? | Targeted practice; controlled preparation; continued activity; reduced intention; avoidance; private or solitary activity | Preserved temporal distinctions and did not treat interview reports as observed longitudinal behaviour |
| Boundary evidence | What challenged a simple testing-to-shame pathway? | Public testing without shame; low score with encouragement; procedural frustration without self-blame; continued movement under lower visibility | Specified theoretical limits and prevented one-directional interpretation |

## ***B3. Illustrative incident trails***

| **Incident** | **Assessment conditions** | **Attribution and scope of judgement** | **Emotional meaning** | **Reported or anticipated orientation** | **Analytic use** |
| --- | --- | --- | --- | --- | --- |
| P2: 800-metre performance during menstruation | Public comparison; valued ranking | Temporary bodily state; performance did not define ability | Anxiety and feeling wronged, not shame | Explanation and targeted running | Public visibility was insufficient for self-devaluation when attribution remained specific |
| P13: first sit-and-reach attempt | Private result; demonstrated stretch; retest offered | Specific and repairable task problem | Concern without loss of face | Practice and retest | Privacy became protective when paired with actionable guidance |
| P1: public pull-up attempt | Public counting; identity-laden teacher comment; peer laughter | Result expanded to gendered and disciplinary identity | Shame-related wish to hide | Concealment and reduced visibility | Teacher talk shifted judgement from performance to person |
| P4: dizziness during 800-metre run | Peer visibility; comment about future medical suitability | Temporary difficulty interpreted as professional inadequacy | Shame/humiliation | Exit and solitary lower-exposure activity | Identity diagnosticity widened the value and scope of failure |
| P8: changed sit-and-reach standard | Criteria changed with limited notice and redress | Problem attributed to procedure rather than ability | Frustration and procedural resistance, not shame | Complaint and stated continuation of usual activity | External attribution redirected negative evaluation towards the assessment |
| P26: process recognition | Teacher prioritised persistence over score | Performance remained bounded and potentially learnable | Encouragement | Later participation not established by the quoted extract | Process recognition altered the immediate meaning of assessment without supporting a behavioural inference |
| P29: profession-relevant guidance | Guidance connected movement with the bodily demands of drawing | Performance remained context-specific and adaptable | Concern without self-devaluation | Stretching and indoor walking | Adaptation to a lower-exposure setting did not necessarily mean withdrawal from movement |

## ***B4. Cross-theme synthesis***

| **Pathway** | **Appraisal/attribution pattern** | **Emotion described in the account** | **Reported or anticipated orientation** | **Evidential boundary** |
| --- | --- | --- | --- | --- |
| Repairable task judgement | Outcome valued and uncertain; cause specific or temporary; credible route to improve | Anxiety, concern, regret | Preparation, guidance seeking, continued participation | Anxiety did not necessarily imply withdrawal |
| Person- or identity-directed judgement | Participant interpreted public or authoritative commentary as extending performance into a judgement of the person or a valued identity | Shame, humiliation, embarrassment, or loss of face, reported separately | Concealment, avoidance, lower-visibility participation | Visibility alone was not sufficient, and not every label was shown to become a stable self-belief |
| System-directed judgement | Cause located in opaque, unstable, or inflexible assessment procedure | Procedural frustration; anger only where supported by the wider account | Complaint or resistance | Neither presented account included an explicit deficient-ability attribution; procedural objections co-occurred with complaint or resistance |

# **Supplementary Appendix C. Anonymised participant profile**

| **Participant** | **University** | **Academic year** | **Major category** | **Gender** | **Secondary-school training or regular exercise** |
| --- | --- | --- | --- | --- | --- |
| P1 | A | First year | Engineering | Man | No |
| P2 | A | First year | Liberal arts | Woman | Yes |
| P3 | A | First year | Economics/management | Man | No |
| P4 | A | First year | Medicine | Woman | No |
| P5 | A | First year | Fine arts | Man | Yes |
| P6 | A | First year | Engineering | Woman | No |
| P7 | A | First year | Liberal arts | Man | Yes |
| P8 | A | Second year | Economics/management | Woman | Yes |
| P9 | A | Second year | Medicine | Man | No |
| P10 | A | Second year | Fine arts | Woman | No |
| P11 | A | Second year | Engineering | Man | No |
| P12 | A | Second year | Liberal arts | Woman | Yes |
| P13 | B | First year | Economics/management | Woman | Yes |
| P14 | B | First year | Medicine | Man | No |
| P15 | B | First year | Fine arts | Woman | No |
| P16 | B | First year | Engineering | Man | Yes |
| P17 | B | First year | Liberal arts | Woman | No |
| P18 | B | Second year | Economics/management | Man | No |
| P19 | B | Second year | Medicine | Woman | Yes |
| P20 | B | Second year | Fine arts | Man | No |
| P21 | B | Second year | Engineering | Woman | Yes |
| P22 | B | Second year | Liberal arts | Man | No |
| P23 | C | First year | Medicine | Woman | No |
| P24 | C | First year | Fine arts | Man | Yes |
| P25 | C | First year | Engineering | Woman | No |
| P26 | C | Second year | Liberal arts | Man | No |
| P27 | C | Second year | Economics/management | Woman | Yes |
| P28 | C | Second year | Medicine | Man | No |
| P29 | C | Second year | Fine arts | Woman | No |
| P30 | C | Second year | Engineering | Man | Yes |

**Note.** The profile summarises participant-reported information to support contextual interpretation, not statistical representativeness. Combinations of characteristics and distinctive incidents may carry local recognition risk; access to more detailed case information is therefore restricted.

# **Supplementary Appendix D. Quotation alignment and translation evidence**

| **Participant** | **Chinesesourceexcerpt** | **Englishtranslationusedinthemanuscript** | **Analyticuse** |
| --- | --- | --- | --- |
| P1 | 自己一个都拉不上去，老师当众说“工科男生体能这么差”，同学还发出笑声。 | I could not do even one pull-up. The teacher said publicly, “An engineering boy with physical fitness this poor,” and some classmates laughed. | Public visibility and identity-laden teacher commentary |
| P2 | 我有一点焦虑，但不是羞耻。只是生理期状态不好，不代表我不行。 | I felt a little anxious, but it was not shame. I was in a poor bodily condition because of menstruation; it did not mean I was incapable. | Anxiety without global self-devaluation |
| P4 | 老师说“这点体能都没有，以后当医生怎么行”，我觉得不是在说跑步，是在说我不适合这个专业。 | The teacher said, “With this little stamina, how will you become a doctor?” I felt he was not talking only about running, but saying I was not suitable for my major. | Person-directed judgement tied to professional identity |
| P6 | 测试后锻炼意向完全为零，看到操场就觉得不舒服。 | After the test, my intention to exercise dropped to zero. Seeing the track makes me uncomfortable. | Avoidance and place-associated discomfort; the extract alone does not establish a specific emotion |
| P8 | 不是觉得羞耻，是觉得标准突然变了，努力变得没有意义。 | It was not that I felt ashamed. The standard changed suddenly, and our effort became meaningless. | Procedural frustration and external attribution rather than shame |
| P13 | 只有老师和我知道结果，所以不觉得丢脸；我知道该练什么。 | Only the teacher and I knew the result, so I did not feel ashamed; I knew what to practise. | Privacy and actionable feedback |
| P26 | 老师说坚持比成绩更重要，第一次在体育测试里觉得被鼓励。 | The teacher said persistence mattered more than the score; it was the first time I felt encouraged in a PE test. | Process recognition and encouragement |
| P29 | 老师说美术生的手部和腰背体能更重要，可以把训练和绘画结合。 | The teacher said fine arts students' hand and lower-back fitness were more important and that training could be connected with drawing. | Profession-relevant, lower-exposure participation |

**Translation note.** The Chinese excerpts are the evidence base for all verbatim quotations reproduced in the article. English wording is meaning-based and retains the evaluative target and action tendency. The translation of 丢脸 as “ashamed” in P13 is contextual rather than a claim that loss of face and shame are universally equivalent. Other descriptions in the findings are paraphrases and are not presented within quotation marks.
